# Chemoorganotrophic Bacteria From Lake Fryxell, Antarctica, Including *Pseudomonas* Strain LFY10, a Cold-Adapted, Halotolerant Bacterium Useful in Teaching Labs

**DOI:** 10.3389/fmicb.2019.00156

**Published:** 2019-02-06

**Authors:** Jennifer M. Baker, Nicole A. Vander Schaaf, Anna M. G. Cunningham, Anna C. Hang, Chelsea L. Reeves, Emily R. Huffman, Carli J. Riester, Michael T. Madigan, W. Matthew Sattley

**Affiliations:** ^1^Division of Natural Sciences, Indiana Wesleyan University, Marion, IN, United States; ^2^Department of Microbiology, Southern Illinois University, Carbondale, IL, United States

**Keywords:** Antarctica, Lake Fryxell, McMurdo Dry Valleys, chemoorganotroph, psychrotolerant, halotolerant, *Pseudomonas*

## Abstract

Lake Fryxell, situated in the McMurdo Dry Valleys of Antarctica, is an intriguing aquatic ecosystem because of its perennial ice cover, highly stratified water column, and extreme physicochemical conditions, which collectively restrict lake biodiversity to solely microbial forms. To expand our current understanding of the cultivable biodiversity of Lake Fryxell, water samples were collected from depths of 10 and 17 m, and pure cultures of eight diverse strains of aerobic, chemoorganotrophic bacteria were obtained. Despite having high 16S rRNA gene sequence similarity to mesophilic bacteria inhabiting various temperate environments, all Lake Fryxell isolates were psychrotolerant, with growth occurring at 0°C and optimal growth from 18–24°C for all isolates. Phylogenetic analyses showed the isolates to be members of six taxonomic groups, including the genera *Brevundimonas, Arthrobacter, Sphingobium, Leifsonia*, and *Pseudomonas*, as well as the family *Microbacteriaceae* (one strain could not reliably be assigned to a specific genus based on our analysis). *Pseudomonas* strain LFY10 stood out as a useful tool for teaching laboratory activities because of its substantial cold adaptation (visible growth is evident in 1–2 days at 4°C), beta-hemolytic activity, and halotolerance to 8.5% (w/v) NaCl. These cold-adapted bacteria likely play a role in carbon mineralization and other nutrient cycling in Lake Fryxell, and their characterization broadens our understanding of microbial biodiversity in aquatic polar ecosystems.

## Introduction

**FIGURE 1:**
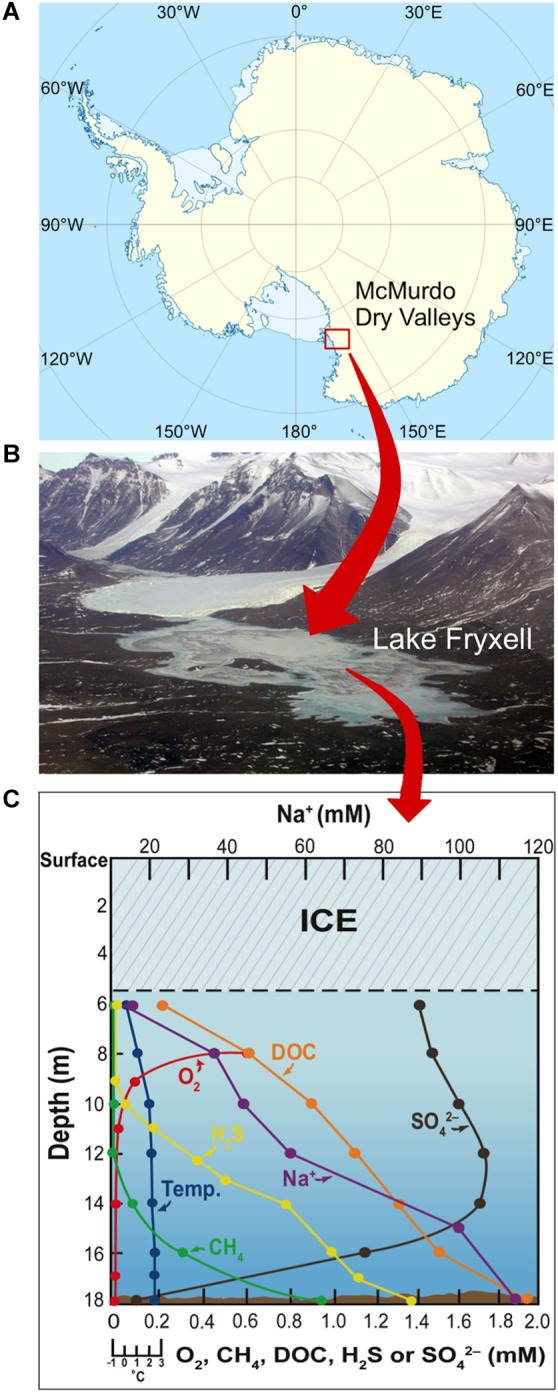
Geomorphology and physicochemistry of Lake Fryxell, Antarctica. **(A)** Site of the McMurdo Dry Valleys of East Antarctica. **(B)** Aerial view of Lake Fryxell in Taylor Valley. **(C)** Physicochemistry of the highly stratified Lake Fryxell water column (adapted from Sattley and Madigan, 2010). DOC, dissolved organic carbon.

The McMurdo Dry Valleys of East Antarctica (Figure 1A) comprise a windswept polar desert that receives minimal annual precipitation (3–50 mm water equivalent per year; Fountain et al., 2009) and consists of nutrient-poor, rocky–sandy soils. The valleys contain several permanently ice-covered lakes that contain a broad diversity of microorganisms adapted to harsh physicochemical conditions, including constant freezing temperatures, nutrient limitation, and low incident light with long periods of almost total darkness during the austral winter. Lake Fryxell, located in Taylor Valley, has a water column of 18 m covered by 5–6 m of ice that prevents wind mixing and limits light penetration (Figure 1B). Beneath the ice cover, Lake Fryxell waters are fresh and supersaturated with trapped gasses, especially O_2_ from phytoplankton photosynthesis (Vincent, 1981; Priscu et al., 1987; Figure 1C). Deeper strata are brackish and anoxic and experience the upward diffusion of dissolved sulfide produced by sulfate-reducing bacteria (Karr et al., 2005; Sattley and Madigan, 2010; Figure 1C). Dissolved O_2_ and benthic sulfide coexist as opposing gradients to produce a biologically active chemocline from 9–10.5 m, wherein numbers of sulfur-oxidizing bacteria peak (Sattley and Madigan, 2006).

Several studies have characterized novel bacterial isolates from Lake Fryxell microbial mats (Van Trappen et al., 2004a,b) and sediments (Sattley and Madigan, 2007, 2010), as well as the water column itself (Jung et al., 2004; Sattley and Madigan, 2006; Sattley et al., 2008). Other studies have employed culture-independent methods to examine these habitats (Brambilla et al., 2001; Karr et al., 2003, 2005, 2006; Kwon et al., 2017). Taken together, these investigations have revealed that extensive microbial diversity exists in Lake Fryxell and that only a handful of species have been isolated and characterized. Of the Lake Fryxell water column isolates, bacteria that grow phototrophically or drive microbial sulfur cycling have been the best characterized, including anoxygenic phototrophs, sulfur-oxidizing bacteria, and sulfate-reducing bacteria (Karr et al., 2003, 2005; Sattley and Madigan, 2006, 2010). By contrast, very few enrichments targeting aerobic, heterotrophic bacteria from the Lake Fryxell water column have been performed.

Although Antarctic lakes exhibit a surprising degree of microbial diversity, we have relatively few isolates from these ecosystems compared to temperate aquatic environments and know little of their genetic or biochemical potential. We have thus employed both defined and complex culture media and specific incubation conditions to enrich for cold-adapted, chemoorganotrophic bacteria from different depths of the Lake Fryxell water column. These enrichments yielded pure cultures of eight diverse strains of bacteria, and here we describe the morphology, physiology, and phylogeny of these isolates with a focus on *Pseudomonas* strain LFY10, a potentially useful strain for teaching laboratories in microbiology.

## Materials and Methods

### Sample Collection and Strain Isolation

Water samples were collected from Lake Fryxell, Antarctica (77°36.630′S, 163°08.853′E) using a peristaltic pump-driven limnological sampling device, as previously described (Sattley and Madigan, 2010; Sattley et al., 2017). Sampled portions of the water column included both the chemocline (10 m) and the anoxic benthos (17 m) of the lake. Primary enrichment cultures were then established at 0, 4, and 8°C at *in situ* pH (pH 7–7.4; Lee et al., 2004) in two ways. The first method was to prepare spread plates of Lake Fryxell water samples inoculated onto R2A medium (Reasoner and Geldreich, 1985). Colonies on these plates that had distinct morphological properties were then successively streaked for isolation on medium R2A at 4°C.

A second enrichment method was employed in which we added 1 ml of lake water to each of several 125-ml Erlenmeyer flasks containing 25 ml of a defined minimal medium containing glucose as sole carbon source (hereafter referred to as “ammonium-glucose medium”), as previously described (Vander Schaaf et al., 2015). The flasks were then incubated on a gently rotating orbital shaker placed inside a cooling box. After two weeks, turbid cultures were transferred to agar plates containing the same ammonium-glucose medium, and pure cultures were obtained by streaking for isolation. Axenic cultures of all bacterial strains were confirmed by microscopic observation and routinely maintained by periodic transfer into tubes of liquid R2A or the ammonium-glucose medium incubated at 4°C.

### Morphology

Cell morphology was determined using a Leica Microsystems Model DM1000 phase-contrast light microscope. Capsule stains were performed using Maneval’s method (Maneval, 1941). Motility was determined by microscopic observation and visual assessment of stab inoculations into semi-solid (0.35% agar) sulfide-indole-motility (SIM) medium (Becton, Dickinson and Company, Sparks, MD, United States; Zimbro et al., 2009).

For transmission electron microscopy (TEM), samples of late logarithmic/early stationary phase cells grown at 4°C were fixed in 2.5% glutaraldehyde in 0.1 M sodium cacodylate buffer, post-fixed in buffered 1% osmium tetroxide containing 0.8% potassium ferricyanide, and stained in 1% aqueous uranyl acetate. The samples were then dehydrated with a graded series of acetonitrile and embedded in EMbed-812 resin. Thin sections (80 nm) were stained with 2% uranyl acetate and lead citrate and imaged using an FEI/Philips CM-10 transmission electron microscope (FEI Company, Hillsboro, Oregon, United States). Samples for scanning electron microscopy (SEM) were fixed in buffered 2.5% glutaraldehyde, post-fixed in 1% osmium tetroxide, dehydrated in a graded ethanol series, and critical-point dried. Specimens were then platinum-coated and cryosamples imaged using an FEI NOVA nanoSEM field emission scanning electron microscope.

### Physiological Studies

A modified liquid R2A medium lacking soluble starch was used to determine the growth temperature range and optima and salinity tolerance of all eight isolates. To establish cardinal growth temperatures, triplicate 10-ml screw-cap tubes containing 3 ml of medium (pH 7.2) were inoculated and incubated for one week at selected temperatures within a range of −2 to 39°C. After a seven-day incubation period, growth was assessed spectrophotometrically using a Spectronic 20D+ (Spectronic Instruments, Inc.), and absorbance readings (OD_540_) were converted into a percentage of the maximum absorbance value. Growth rate experiments were conducted in a similar fashion, with absorbance measurements taken a total of ten times at two- to three-day intervals over a period of 19 days.

To determine salinity tolerances, triplicate cultures of each isolate were established in liquid R2A media supplemented with NaCl (0–12% [w/v], depending on the strain) and incubated at 15°C. Increments of 2% NaCl were used to determine approximate salinity ranges, and relevant concentrations in 0.25% NaCl increments were further tested to define each strain’s salinity tolerance. Cultures were incubated for up to one month, and growth was evaluated by periodic visual observations of turbidity and confirmed by microscopic observation.

To investigate carbon source utilization, culture tubes containing the defined ammonium-glucose medium were prepared as previously described (Vander Schaaf et al., 2015), with a variety of individual filter-sterilized carbon sources substituted for glucose. Duplicate tubes were inoculated and incubated at 15°C for 10 days. After incubation, growth was assessed spectrophotometrically (OD_540_), and absorbance readings were assigned +/− symbols to denote growth intensity and simplify presentation.

Biochemical assays for the activity of specific catabolic enzymes for cysteine, tryptophan, citrate, phenylalanine, urea, gelatin, and starch were performed for all strains as previously described (Vander Schaaf et al., 2015). Likewise, the potential for anaerobic growth was tested in all strains using anoxic media containing 10 mM (f.c.) of either dimethyl sulfoxide, sodium nitrate, or sodium fumarate as alternative electron acceptors (Vander Schaaf et al., 2015). Anoxic conditions were achieved in screw-cap tubes completely filled with medium reduced with 0.3 mM (f.c.) sodium sulfide and containing resazurin (1 mg/l) as a redox indicator. Hemolytic activity was assayed for strain LFY10 by inoculating sheep blood agar (SBA) plates and incubating at 4 and 22°C. Following incubations of five days and 48 h, respectively, colonies of fully grown cultures were visually inspected to determine hemolysis.

### Phylogenetic Analyses

Small subunit (16S) rRNA genes were PCR amplified from genomic DNA isolated from mid-exponential phase cells using bacterial primers 8F (5-AGAGTTTGATCCTGGCTCAG-3) and 1525R (5-AAGGAGGTGATCCAGCC-3) and the following cycling parameters: initial denaturation at 94°C for 30 s; 30 cycles of 30 s at 94°C, 30 s at 54°C, and 60 s at 72°C; and a 10-min extension at 72°C. DNA-free controls were included in all PCR reactions, and 16S rRNA gene amplification products were confirmed to be of proper size (∼1500 bp) by comparison to a 1 Kb DNA ladder (Invitrogen, Carlsbad, CA, United States). Bacterial DNA from each strain was purified from gels using the QIAquick Gel Extraction Kit (QIAGEN Sciences, Germantown, MD, United States) according to the manufacturer’s instructions. Sequence fragments of purified 16S rRNA genes were assembled, and multiple sequence alignments were generated using UGENE version 1.11.0 (Okonechnikov et al., 2012), MUSCLE (Edgar, 2004) and MEGA 7 (Kumar et al., 2016). Positions with gaps or missing data were eliminated from the analysis. 16S rRNA sequences of all other strains used in phylogenetic analyses were obtained from the National Center for Biotechnology Information (NCBI) GenBank database.

Phylogenetic relationships were determined using BLAST (Johnson et al., 2008), the Ribosomal Database Project (Cole et al., 2009), and the List of Prokaryotic Names with Standing in Nomenclature (Parte, 2018). Methods of phylogenetic tree construction are described in their legends. Sequence identity percentages were generated using NCBI’s megaBLAST algorithm (Camacho et al., 2009).

16S rRNA gene sequences from all eight strains of Lake Fryxell bacteria were deposited into GenBank under the accession numbers shown in the phylogenetic trees. Cultures of the Lake Fryxell isolates were preserved at −80°C in growth medium containing 10% (w/v) dimethyl sulfoxide (DMSO) and are available to qualified researchers from the corresponding author upon written request.

## Results

### Enrichment, Isolation, and Morphology

Following an initial incubation period of two (at 8°C) to five (at 0°C) weeks, depending on temperature, primary enrichment cultures of Lake Fryxell water samples spread on plates of R2A medium yielded a diversity of pigmented colonies ranging from pale yellow to reddish-orange. By contrast, liquid enrichment cultures in ammonium-glucose medium produced glossy, unpigmented colonies. Colonies from primary enrichment cultures that showed morphological variation and rapid growth were selected for further study. A total of eight strains of cold-active, aerobic, chemoorganotrophic bacteria were isolated from the Lake Fryxell water samples: five strains from a depth of 10 m and three strains from a depth of 17 m (Table 1). Numbers in strain designations denote the sampling depth (m) from which each strain was isolated.

**Table 1:** Major properties of eight bacterial strains isolated from Lake Fryxell, Antarctica^∗^.

Property	LFY10	FO10	FS17/FL10	FT17	FY10/FYS10	FO17
Colony Pigmentation	None	Bright yellow	None	Pale yellow	Yellow	Red to orange
Morphology	Rod	Rod	Rod	Rod	Coccobacillus	Straight-to-curved rod
Capsule	+	+	+	+	+	+
Gram stain	−	−	−	−	+	−
Cell size (l × w, μm)	1.7–3.7 × 1–1.2	1.8–6 × 0.8–0.9	1.4–3.5 × 0.7–0.8	1.8–2.7 × 0.6–0.7	1.3–2.2 × 1.2–1.4	1.6–3.8 × 0.6–0.8
Motility	++	−	+/(+)	−	−	−
Optimal Temp. (°C)	24	20	20	20	18	20
Temp. Range (°C)	−2 to 36	−1 to 34	−1 to 35	0–28	0–29	0–28
Salinity range (% NaCl)	0–8.5	0–2	0–4.25	0–5.25	0–10/0–9.5	0–11

*^∗^Motility was determined by microscopic observation and the use of sulfide/indole/motility (SIM) medium. Strains that share a column showed identical results except where separated by a forward slash. Gram reactions were determined by staining and verified using the KOH test (Buck, 1982). Numbers in strain designations indicate sampling depth (m) from which each strain was isolated.*

**FIGURE 2:**
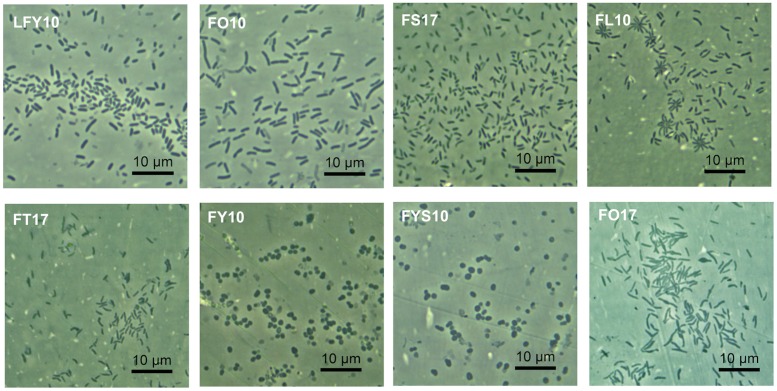
Cellular arrangement and morphology of Lake Fryxell isolates. Photomicrographs (1000× total magnification) show the eight bacterial Lake Fryxell isolates obtained during this study. Inset text denotes strain designation.

When grown on plates of medium R2A, colonies of strains FY10 and FYS10 were yellow, while those of strain FO10 were bright yellow, strain FT17 pale yellow and translucent, strain FO17 reddish-orange, and strains FS17, FL10, and LFY10 colorless and opaque (Table 1). Most strains produced circular, shiny colonies with entire margins, but colonies of strain LFY10 had undulated margins and a mucoid appearance. Cells of strains FY10 and FYS10 were coccobacilli; cell morphologies of all other strains were rods of various sizes (Table 1 and Figure 2).

### Phylogeny

**FIGURE 3:**
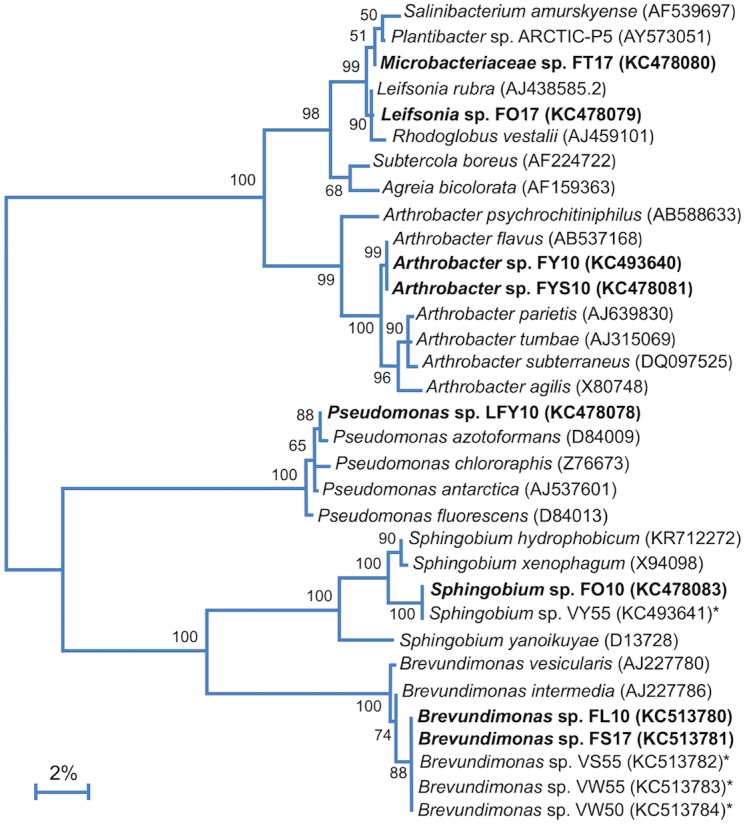
16S rRNA phylogenetic analysis of the Lake Fryxell bacterial isolates (bold). 16S rRNA gene sequences from Lake Fryxell isolates and type strains of related species were aligned using Muscle (*n* = 1311 in final data set). The tree was constructed in Mega7 (v. 7.0.26) using the Maximum Likelihood method and the Jukes-Cantor model. 500 bootstrap replicates were obtained to determine validity (values shown at branch points indicate the percentage of replicates in which the node was observed). Asterisks (^∗^) indicate isolates from nearby Lake Vanda described in our previous work (Vander Schaaf et al., 2015). GenBank accession numbers are shown in parentheses. The scale bar indicates a distance equivalent to 2 nucleotide changes per 100 nucleotides.

Phylogenetic studies revealed that the Lake Fryxell isolates belonged to five distinct phylogenetic clades (Figure 3). BLAST analyses of 16S rRNA gene sequences indicated that strains FT17 and FO17 were members of the family *Microbacteriaceae*. Both of these strains were closely related (≥99% 16S rRNA gene sequence identity) to *Leifsonia rubra*, but strain FT17 also clustered tightly with species of *Plantibacter* and *Salinibacterium*. Strains FY10 and FYS10 aligned closely with *Arthrobacter flavus*, sharing >99% 16S rRNA gene sequence identity. Strains FS17 and FL10 showed 100% sequence identity to each other and also to three other Antarctic strains of the genus *Brevundimonas* (strains VS55, VW55, and VW50; Figure 3). Strain FO10 grouped within the genus *Sphingobium*, having an identical 16S rRNA gene sequence to Antarctic *Sphingobium* sp. VY55 and 99% identity to *Sphingobium xenophagum* (Pal et al., 2006).

**FIGURE 4:**
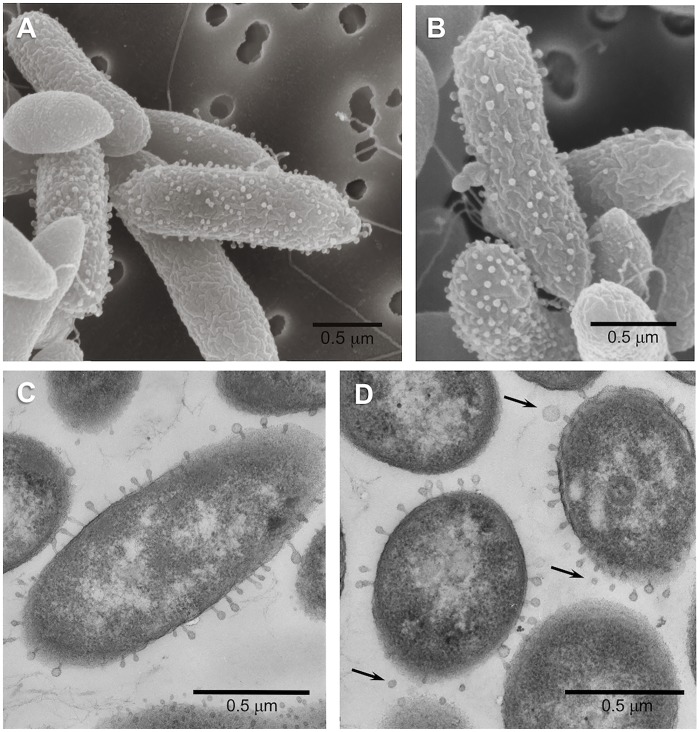
Scanning **(A,B)** and transmission **(C,D)** electron micrographs of cells of *Pseudomonas* strain LFY10 grown at 4°C. Surface blebbing can be seen in both SEM and TEM imaging and are visible only on some cells. These structures, some of which appear to be completely detached (arrows, **D)**, are likely membrane vesicles produced by this Gram-negative bacterium. Note also the presence of polar flagella in the SEM images.

**FIGURE 5:**
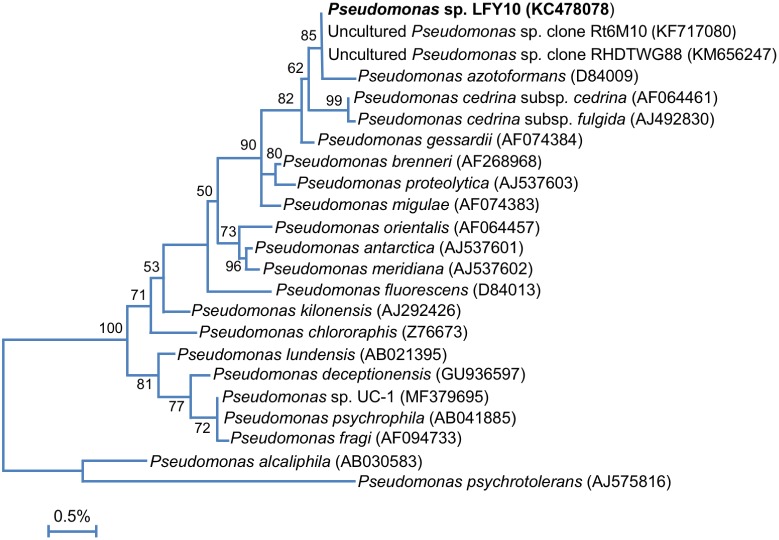
16S rRNA phylogenetic analysis of strain LFY10 and related *Pseudomonas* strains. 16S rRNA gene sequences from strain LFY10 and other *Pseudomonas* species were aligned using Muscle (*n* = 1387 in final data set). The tree was constructed as for Figure 3. GenBank accession numbers are shown in parentheses; type strains of each species were used unless otherwise noted. The scale bar indicates a distance equivalent to 0.5 nucleotide changes per 100 nucleotides.

Phylogenetic analysis of strain LFY10, a Gram-negative rod (Figure 4), showed it to group within the *P. fluorescens* clade of the genus *Pseudomonas* (Figure 3, 5). The closest well-characterized relative of strain LFY10 was *Pseudomonas azotoformans* (>99% identity). Other relatives of strain LFY10 included several Antarctic *Pseudomonas* species, including *P. meridiana*, *P. proteolytica*, and *P. antarctica*, all isolated from Wright Valley cyanobacterial mat samples and glacial streams (Reddy et al., 2004), as well as the more distantly related *Pseudomonas* sp. UC-1, isolated from South Pole ice samples (Madigan et al., 2017).

### Physiology

All strains showed robust growth on R2A medium. None of the isolates grew anaerobically, either by fermentation or anaerobic respiration of alternative electron acceptors (nitrate, fumarate, and DMSO). Carbon source utilization profiles varied among the Lake Fryxell isolates, but metabolic versatility was a general trend (Table 2). Yeast extract supported good growth of all Lake Fryxell isolates, and all strains were able to use acetate, pyruvate, and lactate. Strain FO17 was the most fastidious isolate, as growth of this strain was poor on any substrate tested except yeast extract.

**Table 2:** Carbon source utilization profile of Lake Fryxell isolates.^∗^

Carbon Source	LFY10	FO10	FS17/FL10	FT17	FY10/FYS10	FO17
***Sugars***						
Glucose	++	(+)	+	+	(+)/+	−
Fructose	+	−	(+)/−	+	+	(+)
Galactose	++	(+)	+	+	+	−
Lactose	−	(+)	(+)	(+)	(+)	−
Sucrose	−	(+)	−	+	+	(+)
Ribose	++	−	(+)	(+)	(+)	(+)
Mannose	+	−	(+)	+	+/++	(+)
Maltose	−	(+)	+	−	−	−
Xylose	−	−	−	−	(+)/−	−
***Alcohols***						
Ethanol	(+)	−	+	−	(+)	−
Propanol	−	−	(+)	−	+/(+)	−
***Fatty and Organic Acids***						
Acetate	++	(+)	+	+	+	(+)
Butyrate	(+)	+	+	(+)	+/(+)	−
Propionate	++	−	−	(+)	+/(+)	(+)
Pyruvate	+	+	+	+	+	(+)
Lactate	++	(+)	(+)	+	++	(+)
Fumarate	++	−	+	−	++/(+)	−
Succinate	++	(+)	+	(+)	+ / (+)	−
Benzoate	−	(+)	−	−	(+)	−
Yeast Extract	++	+	++	++	++	++

*^∗^Strains that share a column showed identical results except where separated by a forward slash. Biochemical assays for utilization of cysteine, tryptophan, citrate, phenylalanine, urea, gelatin, and starch were negative for all strains except strain LFY10, which was positive for citrate utilization and gelatinase. Growth was assessed as the difference of the time zero absorbance (OD_540_) from the final absorbance using the following scale: ++, Δ OD_540_ > 0.15; +, Δ OD_540_ 0.051 − 0.150; (+), Δ OD_540_ 0.021 − 0.050; −, Δ OD_540_ 0 − 0.02. Carbon sources had the following final concentrations: Yeast extract, 0.1% (w/v); benzoate, lactose, 3 mM; maltose, sucrose, fructose, glucose, galactose, butyrate, succinate, 5 mM; all others, 10 mM.*

**FIGURE 6:**
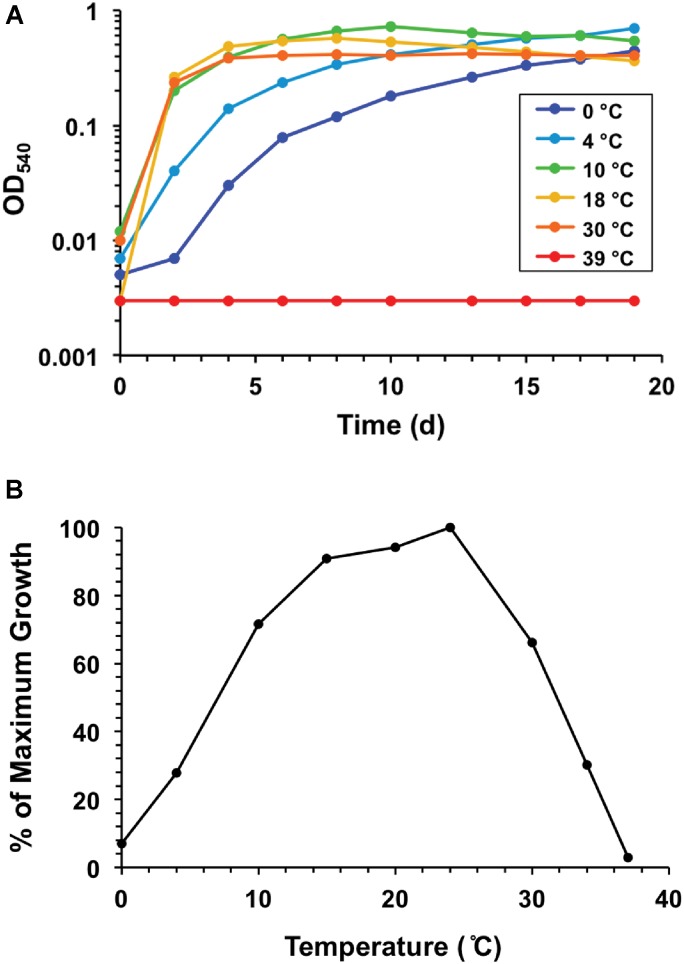
Growth response to temperature for strain LFY10. **(A)** Growth of strain LFY10 over a range of temperatures. Strong growth at low temperatures (0, 4, and 10°C) in combination with no growth at 39°C demonstrates clear cold adaptation. **(B)** Expression of growth rate data as percent maximum growth. The temperature supporting the highest cell density after one week of incubation was assigned a value of 100, and the percentage of maximum growth was calculated for each remaining temperature. Slow growth at −2°C was observed (data not shown). Of the eight Lake Fryxell isolates obtained in this study, strain LFY10 showed the fastest growth rate at 0°C and the widest temperature range for growth (Table 1).

Temperature ranges and optima for the eight Lake Fryxell strains were similar, but not identical (Table 1). The most cold-tolerant isolate, strain LFY10, also displayed the widest temperature range, with slow growth occurring down to −2°C and a maximum growth temperature of 36°C (Figure 6). Although all isolates grew at the near-freezing *in situ* Lake Fryxell temperatures (Figure 1), the optimum growth temperature for all strains was near 20°C; thus, all strains exhibited a psychrotolerant rather than psychrophilic phenotype (Table 1).

None of the isolates required NaCl for growth, but several strains showed significant salt tolerance. Strains LFY10, FY10, FYS10, and FO17 all grew at 8% NaCl (Table 1), and the addition of low levels (up to 1%, w/v) of NaCl stimulated their growth (data not shown). Strain FO17 showed the greatest halotolerance, as it grew in medium containing up to 11% (w/v) NaCl. Strain FO10 was the most sensitive to salt, with no growth occurring above 2% (w/v) NaCl.

### Specific Features of *Pseudomonas* Strain LFY10

Strain LFY10 was studied in more detail because it was the most metabolically diverse of all isolates, showing the strongest growth on the greatest number of carbon sources (Table 2) and growing rapidly at 0°C (Figure 6A). Strain LFY10 was unique in biochemical tests, as it was the only isolate to show enzymatic activity for catabolism of citrate and gelatin. In addition, when incubated on blood agar plates, strain LFY10 showed beta-hemolysis at both 4 and 22°C (data not shown).

Electron micrographs of both ultrathin sections and intact cells of strain LFY10 revealed an unusual morphological feature. By TEM, small “bleb-like” structures could be seen on the surface of some but not all cells of strain LFY10 and were visible on both cross-sectioned and longitudinally sectioned cells (Figure 4). Some of these protuberances appeared to have a “head and tail” morphology reminiscent of attached bacteriophages. However, due to their inconsistent size and shape (and other reasons to be discussed below), it is more likely that these structures are membrane vesicles emerging from the outer membrane of this Gram-negative bacterium.

## Discussion

Phylogenetic analyses showed the Lake Fryxell strains to lie within the families *Sphingomonadaceae*, *Micrococcaceae*, *Microbacteriaceae*, *Caulobacteraceae*, and *Pseudomonadaceae*. These families contain predominantly terrestrial and aquatic bacteria, along with outliers of clinical significance. Of note was the isolation of two strains of *Arthrobacter* (strains FY10 and FYS10), a genus primarily known for inhabiting soil. At least nine other recently described Antarctic species were assigned to this genus, including isolates from Wright Valley cyanobacterial mats (*A*. *flavus* and *A*. *roseus*), aquatic sediments (*A*. *antarcticus* and *A*. *ardleyensis*), soil (*A*. *cryotolerans*, *A*. *gangotriensis*, and *A*. *livingstonensis*), penguin guano (*A*. *psychrochitiniphilus*), and seawater (*A*. *kerguelensis*) (Reddy et al., 2000, 2002; Gupta et al., 2004; Chen et al., 2005; Wang et al., 2009; Pindi et al., 2010; Ganzert et al., 2011a). *Arthrobacter* strains FY10 and FYS10 showed close phylogenetic alignment (>99% identity) to Wright Valley isolate *A. flavus* and also had a similar phenotype. The Lake Fryxell *Arthrobacter* strains and *A. flavus* are all non-motile coccobacilli (Figure 2) with distinctive bright yellow pigmentation, and growth occurs within similar temperature and salinity ranges (Reddy et al., 2000). Interestingly, however, *Arthrobacter* strains FY10 and FYS10 showed considerably higher metabolic flexibility compared to *A. flavus*, a bacterium that utilizes only sorbitol as a carbon source (Table 2; Reddy et al., 2000).

*Brevundimonas* strains FL10 and FS17 had 16S rRNA gene sequences identical to three strains of *Brevundimonas* characterized in our previous study of Lake Vanda (Figure 3), an ice-covered lake located in Wright Valley, which lies adjacent to Lake Fryxell (Vander Schaaf et al., 2015). All five of these Antarctic strains are closely related to the temperate aquatic species *Brevundimonas intermedia* and *B*. *mediterranea* (Poindexter, 1964; Abraham et al., 1999; Fritz et al., 2005). Despite having identical 16S rRNA sequences, strains FL10 and FS17 exhibited differences in fructose utilization and motility. Moreover, these strains were isolated from depths in Lake Fryxell having different physicochemical conditions, with strain FL10 originating from much lower dissolved sodium and sulfide concentrations at 10 m than the brackish, sulfide-rich waters from the strain FS17 isolation depth of 17 m. Species of *Brevundimonas* have a widespread and diverse aquatic distribution, especially in marine habitats, and the isolation of strains FL10 and FS17 from a coastal and brackish Antarctic lake is thus consistent with this generalization (Fritz et al., 2005; Ryan and Pembroke, 2018).

Two strains from the family *Microbacteriaceae* were isolated from Lake Fryxell. Phylogenetic analyses of one of these isolates, designated *Microbacteriaceae* sp. FT17, revealed closely related species from three different genera: *Leifsonia rubra*; *Plantibacter* sp. ARCTIC-P5 (from an unpublished study of Arctic bacteria); and *Salinibacterium amurskyense*, a halotolerant marine bacterium from the Sea of Japan (Han et al., 2003). A marine origin seems likely for strain FT17, but it is unclear whether it represents a novel species. Another isolate of the family *Microbacteriaceae*, which we designated *Leifsonia* sp. FO17, was morphologically and phylogenetically similar to *Leifsonia rubra*; both bacteria produce bright red-to-orange colonies, have a curved-rod morphology, stain Gram positively, and are non-motile. Interestingly, of 17 reported *Leifsonia* species, six (including *L. rubra*) are either psychrotolerant or psychrophilic and originate from Antarctica, with *Leifsonia pindariensis*, isolated from a Himalayan glacier in India, as the sole exception (Reddy et al., 2003, 2008; Pindi et al., 2009; Ganzert et al., 2011b).

As previously described in our study of Lake Vanda water column isolates (Vander Schaaf et al., 2015), *Sphingobium* sp. FO10 was phylogenetically identical to *Sphingobium* sp. VY55 and closely related to *Sphingomonas* spp. Ant17 and BP-7 (Sakai et al., 2007). Although *Sphingobium* spp. VY55 and FO10 share the same optimum temperature and bright yellow pigmentation, strain FO10 showed lesser halotolerance and a slightly wider temperature range than its Lake Vanda counterpart (Vander Schaaf et al., 2015).

Phylogenetic analyses clustered *Pseudomonas* sp. LFY10 within the *P. fluorescens* group of the genus *Pseudomonas* (Figure 5). Under our culture conditions, colonies of strain LFY10 were opaque and did not produce the signature fluorescent yellow pigmentation exhibited by many species in the *P. fluorescens* group. The fluorescent pigments, called pyoverdines, specifically chelate ferric (Fe^3+^) iron and play a key role in Fe^3+^ scavenging during iron-limiting conditions (Ringel and Brüser, 2018). Although it may be that genes for pigment biosynthesis simply were not expressed under the culture conditions employed, another explanation is that strain LFY10 may not have the genetic capacity to produce these pigments. Levels of dissolved iron (Fe^2+^) in Lake Fryxell are approximately 0.2 μM L^−1^ at 10 m (Lee et al., 2004). Therefore, strain LFY10 could presumably obtain iron more efficiently by importing Fe^2+^ through a cation transporter than by sequestering Fe^3+^ through the production of fluorescent siderophores, potentially rendering genes for pyoverdine biosynthesis expendable.

A megaBLASTn search revealed that two 16S rRNA gene sequences identical to that from *Pseudomonas* strain LFY10 had been previously identified in two dissimilar studies, one analyzing the root microbiota of *Vitis vinifera* from an Argentinian vineyard (Salomon et al., 2013) and the other an uncultured clone from an unpublished study in Arunachal Pradesh, India (Figure 5). Strain LFY10 also aligned closely with *P. azotoformans*, a mesophilic pathogen of rice and other cereals (Fang et al., 2016), as well as the aforementioned aquatic and cold-adapted Antarctic species *P. antarctica*, *P. meridiana*, and *P. proteolytica* (Reddy et al., 2004). With growth temperatures ranging from −2 to 36°C and an optimum growth temperature of 24°C (Table 1 and Figure 6), the psychrotolerant Arctic isolate *P. psychrophila* (Yumoto et al., 2001) most closely matches the growth temperature response of strain LFY10.

Considering the physiological and phylogenetic characteristics of the bacteria isolated and described in this study, as well as the fact that Lake Fryxell is situated fewer than 10 km inland from the sea, it seems probable that at least some of these strains have a marine origin. With the possible exception of strain FO10, which shows little halotolerance, these bacteria may be derived from marine microorganisms that were deposited into Lake Fryxell, either by past flooding of Taylor Valley or aerosolization, and subsequently adapted to a planktonic lifestyle in an ice-sealed, stratified water column.

In addition to its physiological properties described above, an unexpected morphological feature of strain LFY10 was the bleb-like structures extending from the outer membrane of some but not all cells (Figure 4). Although the extensions had a phage-like morphology, closer inspection revealed that their dimensions and overall shape varied considerably. The structures had diameters ranging from 30 to 100 nm, and therefore, with such variation, it is unlikely that they were bacteriophages or particles of some other gene transfer agent, which have a defined and constant size (Solioz and Marrs, 1977; Lang et al., 2013, 2017). In addition to this, cultures of strain LFY10 grew robustly upon repeated transfer in both liquid and solid culture media—showing no universal virion-coating of cells in electron micrographs or other signs of phage infection (such as cell lysis)—and produced no detectable free viral particles.

It is likely that the observed surface structures on cells of strain LFY10 were membrane vesicles (MVs). The production of MVs is widespread among microorganisms, discovered first in Gram-negative bacteria and since identified in species from all three domains of life (Deatherage and Cookson, 2012; Anand and Chaudhuri, 2016). *Pseudomonas fluorescens*, a close relative to strain LFY10, produces MVs that are formed when the outer membrane of the cell wall bulges outward, fills with periplasm, and then pinches off to form a spherical vesicle (Guyard-Nicodème et al., 2007; Anand and Chaudhuri, 2016), a process that is consistent with our observations of strain LFY10. In addition, MVs vary in size from 20 to 250 nm (Beveridge, 1999; Nevot et al., 2006; Anand and Chaudhuri, 2016; Pathirana and Kaparakis-Liaskos, 2016), dimensions that correspond with the diameters of the structures extending from the cells of strain LFY10 (Figure 4).

Many Gram-negative bacteria shed MVs during the course of normal growth (Beveridge, 1999; Hagemann et al., 2014; Pathirana and Kaparakis-Liaskos, 2016). In the pathogen *Pseudomonas aeruginosa*, MVs play key roles in biofilm formation and pathogenesis (Park et al., 2014; Metruccio et al., 2016). However, in several cold-adapted Antarctic bacteria, including strains of *Pseudomonas syringae*, *Shewanella vesiculosa*, *Pseudoalteromonas* sp. M4.2, *Psychrobacter fozii*, and *Marinobacter guineae* (Frias et al., 2010; Pérez-Cruz et al., 2013, 2015; Kulkarni et al., 2015), vesicle shedding is a reaction to stress through SOS response activation and can be triggered by conditions such as extreme temperature or hyperosmolarity (Guyard-Nicodème et al., 2007; Frias et al., 2010; Metruccio et al., 2016).

Some species of cold-adapted bacteria also produce extracellular polymeric substances (EPS), which form a thick capsular layer that yields mucoid colonies (Frias et al., 2010), a feature displayed by strain LFY10. In accordance with observations of other Antarctic bacteria made by Frias et al. (2010), electron micrographs of cells of strain LFY10 showed putative MVs in various stages of formation, including several that were detached and spherical (Figure 4D). Membrane vesicles, which embed themselves in EPS, have been shown to be an integral part of this capsular layer in cold-adapted microorganisms, such as *Pseudoalteromonas antarctica* (Nevot et al., 2006). While the EPS in the capsular layer have been implicated in functions such as cryoprotection (Arctic marine bacterium *Colwellia psychrerythraea* 34H) and trace metal binding (Antarctic marine *Pseudoalteromonas* spp.), the role of MVs in the capsular layers is less understood (Marx et al., 2009; Ewert and Deming, 2011; Carillo et al., 2015; Casillo et al., 2018; Tribelli and López, 2018). Proposed functions for MVs in capsular layers include intracellular protein exchange, cell-cell signaling, or survival functions similar to those of the exopolymeric capsular components, but remain unclear without further study (Frias et al., 2010).

A final notable feature of *Pseudomonas* strain LFY10 is that it exhibits a number of ideal characteristics for use as a model cold-active bacterium in microbiology teaching labs. First, it is an excellent substitute for more commonly used psychrotolerant bacteria. For example, species of *Listeria* are often used to demonstrate the growth of microbes at low temperature. However, these species often take up to a week to show visible growth on tryptic soy agar (TSA) plates incubated at refrigerator temperatures (4–5°C). By contrast, strain LFY10 produces visible colonies within 24–48 h at 4°C on the same medium. In addition to growth temperature characteristics, strain LFY10 may be used to demonstrate halotolerance, as it is capable of growth in TSA containing up to 8.5% (w/v) NaCl (a demonstration commonly performed in teaching labs; data not shown) and has been shown to grow at even higher salinities in defined media (Heinzelmann et al., 2015a,b). Moreover, growth of strain LFY10 on blood agar plates may also be used to demonstrate beta-hemolysis, as colonies of this bacterium produce a characteristic clearing of red blood cells when grown on this medium (data not shown). Finally, strain LFY10 does not grow at 37°C (approximate human body temperature), and close relatives of this strain are of environmental or marine origin and are not reported to cause pathogenesis in mammals, minimizing the biosafety concerns that arise when using certain other *Pseudomonas* spp. (e.g., *Pseudomonas aeruginosa*) in a teaching lab setting.

## Conclusion

In conclusion, with its permanent ice cover, seasonal swings of constant darkness or daylight, steady and near-freezing temperatures, and water column that shifts from hyperoxic to anoxic and from freshwater to brackish, the physicochemical conditions of Lake Fryxell have selected for a diversity of hardy microorganisms. Harboring exclusively microbial species, Lake Fryxell has become a favored habitat for studying microbial nutrient cycling in an ice-capped, polar environment. The collective ability of these strains to catabolize a wide range of carbon sources, including sugars, alcohols, and fatty and organic acids (Table 2), suggests that such nutritional versatility provides a fitness advantage and may catalyze dissolved organic carbon (DOC; Figure 1) turnover in the ice-sealed Lake Fryxell water column. The wide growth temperature range of the bacteria described here, including an ability to grow at 0°C, and their impressive metabolic versatility could signal their importance as key contributors to carbon turnover in this challenging aquatic ecosystem.

## Data Availability Statement

The datasets generated for this study can be found in the GenBank nucleotide repository^1^ using either the strain names or accession numbers highlighted in Figure 3.

## Author Contributions

NVS, AC, AH, CJR, CLR, EH, and WMS performed laboratory experiments. JB wrote the initial manuscript draft. WMS designed the experiments and supervised the research. MM wrote the grant proposal that funded sample collection, and WMS wrote grant proposals to support laboratory investigations. WMS and MM edited the final version of the submitted manuscript.

## Conflict of Interest Statement

The authors declare that the research was conducted in the absence of any commercial or financial relationships that could be construed as a potential conflict of interest.

## Footnotes

1 https://www.ncbi.nlm.nih.gov/nucleotide/
